# Critical appraisal of major depression with suicidal ideation

**DOI:** 10.1186/s12991-019-0232-8

**Published:** 2019-05-31

**Authors:** Maurizio Pompili

**Affiliations:** Department of Neurosciences, Mental Health and Sensory Organs, Suicide Prevention Center, Sant’Andrea Hospital, Sapienza University of Rome, Rome, Italy

## Abstract

**Background:**

Regardless of its nature, suicidal ideation, in the absence of another diagnosis, is quintessentially associated with major clinical depression. Although for the characteristics of being depressed it is reasonable to have some wish to die, there is no real attempt to understanding the suicidal mind. Clinicians are therefore often inclined to consider suicidal ideation a symptom of major depression. Yet, most depressed patients do not die by suicide, and many of them never experience suicidal ideation even in the most severe depressing scenario. At a closer look, when one works with suicidal individual, suicide appears complex and not line with the obsolete medical model. There are often warning signs for suicide, and suicidal individuals experience mental pain as a common denominator of many adverse events.

**Case presentation:**

A case report of an entrepreneur with no previous psychiatric history describes the process of meditating suicide as a dimension overlapping the depressive disorder. Details of how this 63-year-old male developed high suicide risk are reported, and clinicians are guided into the understanding of suicide risk.

**Conclusions:**

Nowadays, clinicians are requested to provide an in-depth investigation into the suicidal mind, an assessment adjunctive to the psychiatric evaluation. A phenomenological approach may be the key to unlock the suicidal mind. Clinicians may use such tool in light of the need for the empathic understanding of human suffering as well as a paradigm shift in the care of suicidal individuals.

## Background

Notwithstanding the strong association between suicidal ideation and depression, it is time to re-consider both major depression and suicide risk. For both scholars and laypeople, depression does result in the wish to die, and clinicians are often inclined to include suicidal ideation as well as a suicidal crisis into the clinical manifestations of major depression. This assumption is reasonable for any patient. An individual who is depressed, with insomnia, anhedonia, and facing dysphoria and overall hopelessness about the future could easily conclude that life is not worth living, especially if a rapid reduction of such misery is not readily available or not possible. Moreover, lack of joy and pleasure, impaired ability to concentrate and unpleasant future expectations, as well as feeling of worthlessness and guilt, are all symptoms that can result in the wish to die. As Esquirol [1] noted, “Suicide presents all the characteristics of insanity of which it is but a symptom.”

Only a minority of depressed patients die by suicide, and a large percentage of severely depressed patients never think about suicide [2]. Although some researchers count the lives of those who die by suicide as part of the burden of depression, recent statistics challenge this view, indicating that more than the half of suicides do not merit a psychiatric diagnosis [3]. Furthermore, The US Centers for Disease Control and Prevention has launched an information campaign to shed light on suicide pointing to contributing factors other than mental illness [4].

Contrary to the obsolete medical model of suicide, pharmacological treatment results in the reduction of suicide risk, and this implies that these agents target the components of the suicidal scenario rather than the psychopathological symptoms of major depression. The evidence supporting this hypothesis suggests that there are two separate dimensions, one involving features of a psychiatric disorder and the other one presenting the characteristics of a suicidal crisis, often overlapping but still distinct. Such a conclusion emerges from various studies, among which, those involving lithium [5], ketamine [6–8] and clozapine [9–12]. Furthermore, some evidence supports the notion that being suicidal may limit response to antidepressant treatment in depressed major affective disorder patients, independent of overall symptomatic severity [13, 14]. Such evidence seems to suggest that depressed, suicidal individual represent a peculiar subgroup of patients that request in-depth clinical observation.

Modern psychiatry now needs a new medical model with clinicians being able to appraise depressed individuals with suicidal ideation critically. Depression per se is not a useful tool for a proper understanding of the complexity of suicide, and suicidal ideation is not a proxy for the diagnosis of major depression. The uniqueness of each patient determines the variability of the threshold for sustaining mental pain, a condition dependent on personal experiences starting from childhood. This mental suffering, which has been shown to share the same neuroanatomical circuits of somatic pain [15], is referred to the hurt, anguish, or psychache that takes hold in the mind. Such negative emotions are tied to thoughts that erode the perspective of future expectations, making the future seem ominous. Efforts to manage psychological pain can last weeks, months and sometimes years and, at times, the suffering overcomes the threshold very rapidly, and suicide occurs.

It is possible that human sadness (such as in response to loss, grief, etc.) shares feature with major depression even in the absence of a validated psychiatric diagnosis. Clinical judgment is required to distinguish between the two entities [16, 17].

In line with this, the fifth edition of the Diagnostic and Statistical Manual of Mental Disorders (DSM-5) states, “Diagnosis of a mental disorder should have clinical utility” but “the diagnosis of a mental disorder is not equivalent to a need for treatment. Need for treatment is a complex clinical decision that takes into consideration symptom severity, symptom salience (e.g., the presence of suicidal ideation), the patient’s distress (mental pain)” and “Clinicians may thus encounter individuals whose symptoms do not meet full criteria for a mental disorder but who demonstrate a clear need for treatment or care. The fact that some individuals do not show all symptoms indicative of a diagnosis should not be used to justify limiting their access to appropriate care” ([18], p. 20).

## Case presentation

Mr. SL is a 63-year-old entrepreneur who has been running his own business for about 30 years, achieving success and admiration from his peers. However, due to the economic crisis, he started facing financial difficulties and had problems in carrying out his business, paying salaries to his long-lasting employees, and supporting his family. After trying many options to get support from banks, he realized that his company was in danger. Because of this situation, he started experiencing sadness, insomnia, loss of appetite, hopelessness, and irritability. He described how he saw no way out, and he described himself as in a tunnel with no real solution to his economic problems. Things got worse as the crisis eroded the money he had saved for emergencies.

He also experienced unimaginable physical suffering with unpleasant sensations at the hypochondrium (the upper part of the abdomen) related to anxiety, and he sometimes had dyspnea. Despite these symptoms, he tried his very best to continue and attend to his work activities. After almost 3 months of feeling depressed, he started thinking about suicide. He reported that suicidal ideation gradually became the companion that could provide help and relief from the pain he was experiencing. Having realized that he could rely on suicide as a way out from his problems, he experienced both the pressure of the precarious economic state and a state of relief when lousy news regarding his debts continued to arrive. He thought that he would not be alive anymore in a week or two. A peculiar aspect of his psychopathological state was an “ossimoric” feature, that is, while he was experiencing the sadness and despair for what has happened to his life, he was still able to enjoy some activities such as maintaining his status and playing at his tennis club, as well as going out for dinner and other leisure activities.

After experiencing depression with suicidal ideation for a while, he then concluded that suicide was the only option left. Although he had spent a pleasant bank holiday, early in the morning on returning to work, he thought that he had to put an end to his life. He went to his office, got his gun and started driving at random for hours. He had left two letters for his children explaining what was behind his choice. He had switched off his cellular phone, and his family lost his track oh him for hours. Just before the moment when he decided to use the gun for killing himself, he thought he wanted to speak to a friend to ask him to support his family. This friend proved to be skilled in maintaining a conversation and supporting the patients’ wish to live. The conversation on the cellular phone helped police to trace the patient. Police officers stopped him and brought to a psychiatrist who diagnosed major depression and the need for psychiatric hospitalization.

However, after a few days, he was able to decide whether to remain or discharge himself. After returning home, his children noticed the poor mental state of their father and sought a consultation with the author. The patient underwent a full psychiatric evaluation, as well as an in-depth assessment of suicide risk, with an analysis of his reasons for living versus his reasons for dying. Although the patient was depressed, an intervention for the treatment of depression would not have provided relief for this man. Lithium was prescribed, in association with small doses of an atypical antipsychotic at night, and regular sessions of psychiatric evaluation combined with sessions of psychotherapy were scheduled. This treatment proved to be of great relief for the patient, and he reported a feeling of being understood by my collaborators and by me. He improved dramatically over 2 months and, despite having the same economic problems that had led him to contemplate suicide; he never reported suicidal ideation again.

## Discussion and conclusions

Suicidal individuals have many unmet needs, and they may not fit into diagnostic categories and may lack a full clinical picture. They should not be left alone with no treatment as if therapeutic options would prove to be of no use.

Psychological pain, as a main ingredient of suicide, is the pain of excessively felt shame, guilt, fear, anxiety, loneliness, and angst. This very human condition points to the fact that the nature of suicide is first mental, meaning that each suicidal drama occurs in the mind of a unique individual [19]. Depressed individuals are suicidal only when negative emotions are so painful that suicide is the only option left and when the suicidal mind is hosted in an individual’s depressed brain. Such individuals conclude that life cannot be accepted with unbearable suffering. Suicide is not, therefore, a specific and narrow symptom of depression. Instead, it is a behavior “combining features of a declaration of war with a petition for bankruptcy” [20].

What emerged from the case reported above is the fact this man was experiencing a narcissistic failure from having his business closed down, and his employees fired. He was between life and death, hoping somebody would rescue him and reduce his suffering. From the clinical picture, there also emerged a feature always traceable in suicidal individuals, that is, ambivalence. He was contemplating suicide, but he also was attached to life’s activities, such as his duties and his family, and he ultimately phoned a friend. This feature can be a crucial element in suicide prevention, providing a period available for rescuing the individual in crisis. During this phase, suicidal individuals often communicate, either covertly or explicitly, their intention to die [21]. The suicidal crisis is also often anticipated or accompanied by three symptoms: anxiety (inner turmoil), agitation and irritability [22], key features also found patients with depressive symptoms during mania [23]. Sleep symptoms are often reported occurring well before the emergence of the suicidal ideation. People contemplating suicide, but experiencing ambivalence, often consider what has been crucial in their lives. They may give away books, jewelers, and symbolic objects to someone who will take care of such things after their death.

From the case report above, we learn that two other essential items were at work in the suicidal mind: hopelessness (such as not having positive future expectations) and dramatic mood changes. Hopelessness has been reported as more indicative than depression in the prediction of suicide [24, 25]. The patient, although continuing to work, saw no future in his activity, dismissing claims for payment as something, which he would not deal with anymore. He also alternated pessimistic thoughts with some recreational activities. Of note is that, before the final decision to die and collecting the gun, he had experienced a good mood and a state of enjoyment (playing golf with friends). Suicidal individuals often switch from sadness to positive and enthusiastic thinking, a feature that has been interpreted with having decided to die by suicide and eliminating the ambivalence. What causes suffering is the ruminations and thoughts that reiterate the failures, the shame, the loss and the rejections (to name just a few) so that imagining the abolition of the flow of thoughts in the conscious mind is seen as the ultimate relief. Clinicians must explore death fantasies in suicidal individuals. When suicide risk is deemed to be high, I always ask if the patients have ever thought about his or her afterlife, that is, the funeral, who will attend, or the reaction of the people who will discover the body. In highly suicidal individuals, such fantasies are reported, whereas they often (although not always) evoke horror if the risk of suicide is low.

Clinicians who consider suicidal ideation to be merely a symptom of depression may miss the rare opportunity to get to know a very private aspect of patients and reduce their suffering. They may even uncover the fact that what they are treating is not necessarily major depression with suicidal ideation but rather severe human sadness emerging with suicidal wishes. This critical distinction may dramatically change the outcome for patients. Treatments and therapeutic options depend on the clinical manifestations. Clinicians may use available treatments for targeting symptoms such as insomnia, agitation, and dysphoria. At the same time, major depression with suicidal ideation may benefit from both pharmacological and non-pharmacological interventions, while not ignoring the motives for wishing to be dead which should be at the center of the psychiatric intervention.

Modern psychiatry needs a better interpretation of suicide risk as compared with old models and reductionist explanations of a complex phenomenon. Rather than confining suicidal ideation to the realm of a symptom, clinicians should relocate such event in the complexity of each human being. Defining it as a symptom inevitably suggests that it is a manifestation of a given disease. We are however dealing with disorders rather than diseases, and suicidal ideation may emerge from the unfortunate combinations of various factors that threaten the stability of an individual. The result of such a state may belong to a different domain than the criteria of major depression. Despite the centrality of perspectives derived from genetics and epigenetics, neurobiology, psychobiology, far from being a manifestation of “normality,” suicide risk is ultimately a manifestation of overwhelming mental pain for which clinicians should be able to provide relief. Such clinical task reveals that suicidal impulses (thoughts and actions) are better understood as a pervasive condition whose roots originate from the internalization of pain-producing inner patterns derived from unsolved past experiences. Such vicissitudes influence proximal risk factors for suicide and exacerbate reactions to present adverse events.

Notions presented in this paper are in line with significant campaigns for preventing suicide, which point to the fact that any single factor rarely causes suicide. Factors can include relationship problems, substance misuse, a recent crisis as well as job, financial or legal stress [4]. Furthermore, recent results highlight the role of childhood traumatic experiences in determining vulnerability to both depression [26] and suicide [27]. Recent findings demonstrated that childhood traumatic experiences negatively influence the outcome of major depression in adulthood [28]. Besides, depressed patients who experienced trauma in childhood may be less likely to respond to treatment and achieve remission [29]. Such evidence goes to show that the focus is the person rather than the disorder and that a comprehensive analysis of both clinical assessments of major depression according to psychiatric criteria as well as an empathic understanding of what energizes mental pain is the key role of anyone who is professionally involved in helping such suicidal individuals.

Clinicians should put themselves into the shoes of the individual with whom they are dealing with. They should discern whether it is major depression or sadness and misery derived from accumulating adverse events. A more phenomenological approach would be of help in assessing the suicidal risk formulation in patients with major depression.

## Data Availability

Not applicable.

## Abbreviation

DSM-5: Diagnostic and Statistical Manual of Mental Disorders, fifth edition
